# A Bird’s-Eye View of Molecular Changes in Plant Gravitropism Using Omics Techniques

**DOI:** 10.3389/fpls.2015.01176

**Published:** 2015-12-24

**Authors:** Oliver Schüler, Ruth Hemmersbach, Maik Böhmer

**Affiliations:** ^1^Institute of Aerospace Medicine, Gravitational Biology, German Aerospace CenterCologne, Germany; ^2^Institute of Plant Biology and Biotechnology, Westfälische Wilhelms UniversitätMünster, Germany

**Keywords:** gravity, plants, systems biology, proteomics, transcriptomics, metabolomics, spaceflight, microgravity

## Abstract

During evolution, plants have developed mechanisms to adapt to a variety of environmental stresses, including drought, high salinity, changes in carbon dioxide levels and pathogens. Central signaling hubs and pathways that are regulated in response to these stimuli have been identified. In contrast to these well studied environmental stimuli, changes in transcript, protein and metabolite levels in response to a gravitational stimulus are less well understood. Amyloplasts, localized in statocytes of the root tip, in mesophyll cells of coleoptiles and in the elongation zone of the growing internodes comprise statoliths in higher plants. Deviations of the statocytes with respect to the earthly gravity vector lead to a displacement of statoliths relative to the cell due to their inertia and thus to gravity perception. Downstream signaling events, including the conversion from the biophysical signal of sedimentation of distinct heavy mass to a biochemical signal, however, remain elusive. More recently, technical advances, including clinostats, drop towers, parabolic flights, satellites, and the International Space Station, allowed researchers to study the effect of altered gravity conditions – real and simulated micro- as well as hypergravity on plants. This allows for a unique opportunity to study plant responses to a purely anthropogenic stress for which no evolutionary program exists. Furthermore, the requirement for plants as food and oxygen sources during prolonged manned space explorations led to an increased interest in the identi-fication of genes involved in the adaptation of plants to microgravity. Transcriptomic, proteomic, phosphoproteomic, and metabolomic profiling strategies provide a sensitive high-throughput approach to identify biochemical alterations in response to changes with respect to the influence of the gravitational vector and thus the acting gravitational force on the transcript, protein and metabolite level. This review aims at summarizing recent experimental approaches and discusses major observations.

## Plant Response to Deviations From the Vertical Position

Gravitropism is defined as the bending of a plant/organ along the direction of the gravity vector. Positive gravitropism describes growth toward the gravity vector, e.g., growth of the root into the soil. Negative gravitropism defines growth opposed to the gravity vector, e.g., growth of the shoot into the air (Frank, 1868). Gravitropic signaling and the role of auxin in gravitropism has recently been reviewed (Lopez et al., 2014; Sato et al., 2015; Zadnikova et al., 2015). In this review we will only briefly discuss the current models of gravitropic responses and focus on the molecular changes measured by omics techniques.

In *Arabidopsis* roots, the root cap, which comprises of four tiers of columella cells and lateral root cap cells (Dolan et al., 1993), is known to be the site of gravity perception. Early decapping experiments showed a loss of the plant’s gravitropic response. The ability to sense alterations of the gravity vector was recovered by regeneration of the root cap (Barlow, 1974). Particularly the inner cells of the second tier of columella cells contribute to root gravitropism as shown by laser ablation experiments (Blancaflor et al., 1998). The gravitropic response, on the other hand, takes place in the elongation zone of the root, physically separated from the site of perception.

In the shoot, mostly coleoptiles and pulvini of monocotyledons and hypocotyledons of dicotyledons were studied in respect to their gravitropic response (Sack, 1991). Genetic studies identified the endodermal cell layer in shoots as statocytes (Fukaki et al., 1998; MacCleery and Kiss, 1999).

Plant gravitropism can be divided into distinct phases: susception, perception, transduction, and response (curvature) (Perbal and Driss-Ecole, 2003; Limbach et al., 2005). Any alteration of the influence of the gravity vector is perceived with the help of specialized, starch-containing organelles, so called statoliths, in gravisensing cells (statocytes), which are sedimenting to the cell’s new physiological bottom (Haberlandt, 1900; Caspar and Pickard, 1989; Kiss and Sack, 1989; Kuznetsov and Hasenstein, 1996; Kiss et al., 1997; Sack, 1997; Kiss, 2000; Morita, 2010). According to the starch-statolith hypothesis, sedimentation of the starch-filled amyloplasts triggers a signal transduction cascade leading to an asymmetric auxin transport and a curvature opposite of the gravitational vector. Additional models have been proposed, including the gravitational pressure hypothesis that is based on density differences and consequently the pressure exerted by the cytoplasm on the plasma membrane (Wayne et al., 1990; Wayne and Staves, 1996) or the tensegrity model, in which the membrane is outstretched on the cytoskeleton backbone of the cell and is in equilibrium between tensile and compressive forces (Ingber, 1997). The latter two models are in accordance with experimental data that still show a gravitropic response in starchless mutants (Caspar and Pickard, 1989). Statolith-dependent and -independent systems might also act in parallel (Perbal, 1999). In a refined model of the statolith hypothesis, it was suggested that not the pressure exerted by statolith sedimentation, but their interaction with membrane-bound receptors activates gravity perception (Braun and Limbach, 2006). In characean rhizoids, graviperception requires the contact of statoliths with membrane-bound receptor molecules rather than tension or pressure exerted by the weight of the statoliths (Limbach et al., 2005). Protein interactions between amyloplasts and membrane receptors might involve components of the TOC (TRANSLOCON OF OUTER MEMBRANE OF CHLOROPLASTS) complex (Stanga et al., 2009; Strohm et al., 2014). Experimental data so far, however, are controversial (Staves, 1997; Staves et al., 1997; Braun et al., 2002; Hou et al., 2003, 2004; Limbach et al., 2005; Valster and Blancaflor, 2008).

A common view is, that the biophysical signal of statolith sedimentation or of changes in cytoplasmic pressure is converted into a biochemical signal (Fasano et al., 2001; Plieth and Trewavas, 2002). Models have been put forward in which the sedimentation of statoliths leads to the activation of mechanosensitive ion channels at the plasmamembrane, endoplasmic reticulum, or at the tonoplast (Sievers et al., 1991; Yoder et al., 2001; Allen et al., 2003; Perbal and Driss-Ecole, 2003). In columella cells, the nucleus and the endoplasmic reticulum (ER) are localized at the proximal side of the root meristem and in the periphery of the cell. This ER, called nodal ER (Zheng and Staehelin, 2001), is thought to be a major reservoir for the second messenger Ca^2+^. Statoliths may induce opening of mechanosensitive ion channels under contribution of the nodal ER and second messenger release (Leitz et al., 2009). Alternatively, statoliths may facilitate the opening of mechanosensitive ion channels under contribution of the cytoskeleton (Sievers et al., 1991; Volkmann and Baluska, 1999; Perbal and Driss-Ecole, 2003). The role of the cytoskeleton is still controversially discussed, but recently the general view is, that the actin cytoskeleton is a negative regulator of root gravitropism (Blancaflor, 2013) and might play a role in fine-tuning the gravitropic response.

Calcium elevations are considered a second messenger of early gravitropic signaling events (Sinclair and Trewavas, 1997; Chen et al., 1999; Chatterjee et al., 2000). The contribution of Ca^2+^ to gravitropism was mainly concluded from inhibitor studies (Belyavskaya, 1996), calcium binding proteins (Stinemetz et al., 1987; Heilmann et al., 2001) or cellular messengers known to be related to Ca^2+^ signaling (Perera et al., 1999). Recent studies on *Arabidopsis* seedlings expressing the luminescent Ca^2+^ reporter Aequorin demonstrated transient increases in [Ca^2+^]_cyt_ during the gravitropic response (Plieth and Trewavas, 2002; Toyota et al., 2008). Two waves of Ca^2+^ oscillations were observed, an initial transient Ca^2+^ increase after 3 s and a more sustained flux after 60 s (Toyota et al., 2008). Further second messengers, including Inositol 1,4,5- triphosphate (IP_3_) (Perera et al., 1999, 2001a; Fasano et al., 2002), protons (Fasano et al., 2001), and reactive oxygen species (ROS; Joo et al., 2001) may also play a role in the gravitropic response. How these secondary messengers interact, their kinetics, and how they establish a response, remains unclear.

Signal transduction eventually leads to the relocalization of auxin carriers (Friml, 2003). Auxin is transported by influx carriers of the AUXIN RESISTANT 1/LIKE-AUX1 (AUX1/LAX) family and the eﬄux carriers of the PIN-FORMED (PIN) family (Friml, 2003). This transport is known as the “Chemiosmotic Hypothesis” (Kleine-Vehn and Friml, 2008) or Soda fountain model (Hasenstein and Evans, 1988). AUX1 and PIN1 contribute to auxin transport from vasculature into root tip through protophloem cells. In *Arabidopsis*, mutation in AUX1 results in severely agravitropic roots due to defects in auxin movements from the root apex to the distal elongation zone (Swarup et al., 2001). In the tip, PIN4 targets auxin to the center of the auxin maximum, which is located in the columella cells within the root cap. PIN3, PIN4, and PIN7 are localized in the columella cells. In this area, AUX1 ensures the uptake of auxin by columella cells, while the PIN proteins mediate eﬄux. PIN3 and PIN7 protein distribution is dependent on the orientation of the root in the gravitational field. When the root is growing vertically, PIN3 and PIN7 are distributed symmetrically in the cell. If there is any deviation from the vertical into the horizontal position, PIN3 and PIN7 relocalize within a few minutes. The eﬄux carriers are then accumulating in the plasma membrane of the cell’s new physiological bottom (Friml et al., 2002a,b; Friml, 2003; Kleine-Vehn et al., 2010). The relocalization of PIN3 and PIN7 are the first steps toward the establishments of a lateral auxin gradient upon gravistimulation (Blancaflor and Masson, 2003). While the total auxin flux in the root stays constant, auxin is redistributed from cells on the upper to the lower side of the root tip within 5 min after a gravitropic stimulus (Band et al., 2012), a timescale that is in accordance with statolith sedimentation and asymmetric changes in root pH and intracellular Ca^2+^-concentration. *pin3pin7* mutants are more agravitropic than *pin3* and *pin7* single mutants, suggesting their functional redundancy. The corresponding auxin gradient is transported basipetally through epidermal and cortical cells of the root cap that express PIN2. This eﬄux carrier transports auxin to the elongation zones of the root, which leads to root curvature (Chen et al., 1999; Ottenschlager et al., 2003).

PIN-FORMED protein abundance and localization at the plasma membrane affects gravitropic response. PIN proteins undergo constitutive endocytotic recycling to different domains at the plasma membrane or via the prevacuolar compartment to the lytic vacuole for degradation (Abas et al., 2006; Kleine-Vehn and Friml, 2008). Modulation of vesicular trafficking affects PIN recycling and gravitropic response (Geldner et al., 2004; Paudyal et al., 2014). Recent results indicate that the polar auxin transport (PAT) mediated by PIN proteins can also be modulated by small secretory peptides called GOLVEN. A reduced concentration of those peptides impairs the formation of auxin gradients during tropic responses (Whitford et al., 2012). The GOLVEN signal specifically modulates PIN2 trafficking. Auxin as well as GOLVEN treatment increase PIN2 levels at the plasma membrane (Paciorek et al., 2005; Whitford et al., 2012). Furthermore, Gibberellic acid (GA) increases the level of PIN auxin transporters at the plasma membrane and promotes asymmetric auxin distribution during gravitropic curvature. A dilution and subsequent reduction in GA leads to an increased concentration of growth repressors of the DELLA protein family, which may reduce cell elongation rate (Band et al., 2012; Löfke et al., 2013).

Posttranslational modifications of PIN proteins additionally affect their role in gravitropism. The Ser/Thr kinase PINOID regulates PIN2-mediated basipetal auxin transport by regulating plasma membrane localization of PIN2 (Sukumar et al., 2009; Huang et al., 2010). PIN3 phosphorylation status can also affect root gravitropism (Ganguly et al., 2012). The D6 PROTEIN KINASE (D6PK) may regulate gravitropism via the phosphorylation status of PINs (Barbosa et al., 2014).

Despite the extensive regulation of PIN2, *pin2* mutants are actually not very agravitropic (Chen et al., 1998; Luschnig et al., 1998; Blakeslee et al., 2007). A triple mutant together with members of the p-glycoprotein (PGP) family of auxin eﬄux transporters, PGP1 and PGP19, however, is severely agravitropic (Blakeslee et al., 2007), indicating functional redundancy in basipetal auxin transport.

The auxin gradient promotes differential cell elongation on opposing sides of the stimulated organ. Auxin is known to promote or to inhibit plant growth in a dose-dependent manner. Growth mediated by auxin is based on the acid-growth theory (Grebe, 2005). Auxin is able to activate proton pumps which lead to the excretion of protons into the cell wall. This acidification may lead to a loosening of the cell wall, allowing the cell to grow and expand. After the growth phase, the cell wall regains stability (Grebe, 2005). The result of an auxin-induced differential cell elongation is a gravitropic curvature.

Auxin can rapidly mediate tropic response on a minute to hour timescale while maintaining stable developmental zonation in the root and then slowly influences size and location of these differentiation zones via the regulation of PLETHORA (PLT) transcription factors (Mahonen et al., 2014). GOLVEN peptides act via positive regulation of PLETHORA transcription factors (Whitford et al., 2012).

Finally, statoliths reposition in the columella cells when the root tip reaches 40°, which leads to the restoration of PIN3/PIN7 localization and symmetric auxin flow, about 100 min after a 90° gravitropic stimulus (Band et al., 2012; Sato et al., 2015). The latter phase of the root gravitropic bending response that can last up to 600 min, is likely orchestrated by newly synthesized target genes of auxin signaling (Band et al., 2012).

## Plants’ Response to a Microgravity Environment

Gravity is a constant factor of life on earth. With the aim to achieve functional weightlessness, a status which is often described as simulated microgravity, different approaches are in use, such as clinostats, random positioning machines as well as magnets for magnetic levitation (Herranz et al., 2013a). The rotation devices are based on the assumption that biological systems need to be exposed to the influence of the gravity vector for a minimal period of time to allow them to adjust to it. If the influence of the gravity vector constantly changes its orientation, the object loses under appropriate simulation conditions its sense of direction and thus shows a behavior similar to the one seen under real microgravity conditions. Real microgravity can be achieved by drop towers, parabolic flights, sounding rockets, satellites, or space stations, like the ISS. Experiment time is limited. Therefore, very little is known of how altered gravity is perceived by the plant and how the system adapts to this new environmental situation. A role for calcium elevations in response to microgravity, as in the response to reorientation of the plant, is still controversial (Häder et al., 2006; Salmi et al., 2011; Hausmann et al., 2014). A transcellular calcium gradient in spores of *Ceratopteris richardii* is reduced within seconds in microgravity, indicating a fast regulation of calcium channels similar to the auxin transport in the root tip during gravity perception (Salmi et al., 2011). In *Arabidopsis* callus cultures, instead, an increase in calcium and ROS was detected in response to microgravity (Hausmann et al., 2014).

Cellular responses that are affected by microgravity include the cell cycle, leading to decreased mitotic index and enhanced proliferation rate in meristematic root cells (Medina and Herranz, 2010). A second major target is the plant cell wall. In rice, a reduced thickness of the cell wall with increased extensibility and elongation in shoots and decreased elasticity in roots was observed (Hoson et al., 2002, 2003; Soga et al., 2003). Also changes in lignin levels in response to altered gravity forces have been observed in some plant species, e.g., mung beans, but not in others, e.g., pine and oat (Cowles et al., 1984). Changes in photosynthesis in response to microgravity, however, are controversial. While a reduction of the light harvesting apparatus and a higher chlorophyll a/chlorophyll b ratio was observed, direct measurements of photosynthetic activity revealed no changes in net photosynthesis, photosynthetic proton flux, and overall quantum yield (Stutte et al., 2006). The dependency of photosynthesis on gas exchange may be one reason for inconclusive results. The lack of convection in microgravity leads to reduced air flow resulting in altered gas exchange and accumulation of volatiles, e.g., ethylene (Porterfield, 2002), and is possibly leading to alterations in photosynthetic activity.

## Expression Changes in Response to a Deviation From the Vertical Orientation

Complete sedimentation of the statoliths in the columella cells requires at least 5 min (Blancaflor et al., 1998; MacCleery and Kiss, 1999). The minimal gravitational stimulus that elicits a response, however, is estimated around 1 min (Blancaflor et al., 1998). Changes in secondary messenger concentration, e.g., IP3, pH, and Ca^2+^, have been observed within this time frame. IP3 for example, is stimulated in gravitropic maize pulvini already after 10 s (Perera et al., 1999, 2001b). Observations of the early changes in gene expression may therefore help to identify missing components that translate the biophysical signal of statolith sedimentation into a biochemical signal (**Table 1**). Gravitational stimulation is generally achieved by one-time reorientation of plants in the gravity vector plane. In one of the earliest studies of *Arabidopsis thaliana* seedlings 39 genes showed an altered abundance after 15 min of gravitational stimulation, increasing to 132 genes after 30 min compared to constant 1 g conditions (Moseyko et al., 2002). Functional gene categories included response to oxidative stress, plant defense, heat shock, ethylene response, and calcium binding. Another study on gene expression changes, this time in root apices, identified gravity-specific gene regulations within 5–15 min of reorientation. A cluster of five genes was induced at least three fold by gravitropic stimulation even within 2 min of treatment (Kimbrough et al., 2004). The identified genes contained members of the auxin responsive family, genes that are induced very rapidly by the application of exogenous auxin (McClure and Guilfoyle, 1989). Taken together, these studies indicate that alterations of the influence of the gravitational vector is perceived as an abiotic stress signal when observed on the whole plant level, while in individual cells of plant organs involved in the gravitropic response, auxin signaling plays a major role in signal transmission.

**Table 1:** Studies that quantified differential expression of genes, proteins, and metabolites in response to a deviation from the vertical orientation.

Plant age^1^	Tissue	Replicates	Method	Duration	DEG/DEP/DEM	Growth hardware	Treatment	Reference
**Transcriptomics**					
3 weeks	*A. thaliana* seedlings	3	8 k array	15, 30 min	15 min: 39 30 min: 132	Petri dish, white light, transfer to dark 16 h prior to treatment	90° (dark)	Moseyko et al., 2002
1 week	*A. thaliana* root apices	2	ATH1	2, 5, 15, 30, 60 min	1730	Petri dish, dark	135° (dark)	Kimbrough et al., 2004
3 days	*B. oleracea* etiolated seed- lings, upper and lower flanks of hypocotyl (1 cm)	3	ATH1	2 h	8	Pipette tips, dark	90° (dark)	Esmon et al., 2006
3 weeks	*O. sativa*, upper and lower flanks of shoot base	3	Rice Genome GeneChip	30 min, 6 h	30 min:167 6 h: 1202	Soil, white light, transfer to dark 16 h prior to treatment	90° (dark)	Hu et al., 2013
4–8 cm stem	*A. thaliana*, upper and lower flanks of inflorescence stems	1	*Arabidopsis* 2	10, 30 min	10 min: 0 30 min: 30	Soil, white light	90° (light)	Taniguchi et al., 2014
4 weeks	*Z. mays*, third aboveground node of shoot	2	RNAseq	–	931	Soil, white light	–	Dong et al., 2013
**Proteomics**					
1 week	*A. thaliana*, root apices	3	2D-GE	30 min, 3 h	16	Petri dish, white light	90° (light)	Kamada et al., 2005
8–10 cm stem	*A. thaliana*, top 4 cm of shoot	3	iTRAQ	2, 4 min	82	Soil, white light	90° (light; 4°C)	Schenck et al., 2013
**Transcriptomics and Metabolomics**					
1 week	*A. thaliana*, whole seedlings	1 n.a.	ATH1 GC-MS	24 h	Genes: 339 Metabolites: 15	Petri dish, white light	90° (dark)	Millar and Kiss, 2013

*^1^At start of treatment; DEG/DEP/DEM: differentially expressed genes, proteins, metabolites; n.a.: information not available; Rep: number of biological replicates.*

The importance of auxin, also with respect to higher plants’ shoot gravitropism, was underlined in at least three transcriptomic approaches. According to the Cholodny/Went hypothesis, an asymmetric auxin transport leads to a curvature in the direction of the gravitational vector (Went and Thimann, 1937). Auxin biosynthesis and signaling transcript levels changed after a deviation from the vertical position or in an already known agravitropic mutant (Esmon et al., 2006; Dong et al., 2013; Taniguchi et al., 2014). While no expression changes were observed after 10 min, at 30 min 30 genes changed in abundance, of which 19 transcripts were auxin responsive genes of the AUX/INDOLE-3-ACETIC ACID INDUCIBLE (IAA) and SMALL AUXIN UPREGULATED (SAUR) families (Taniguchi et al., 2014). Transcript analysis of plants of *Zea mays* wildtype and *zmla1* mutant, a homolog of the *Arabidopsis* LAZY1 gene and an agravitropic mutant regulating PAT, identified 931 alterations in transcript expression. GO annotation of the altered genes and localization studies suggested a function for LAZY1 in auxin signaling and translocation of auxin exporters (PIN proteins) (Dong et al., 2013). When the focus was set on changes in gene expression in opposing flanks of graviresponsive tissue, e.g., hypocotyl, shoot base or inflorescence stems, a role for auxin in these responses became particularly clear (Esmon et al., 2006; Hu et al., 2013; Taniguchi et al., 2014). Two hours of gravitropic and phototropic stimulation of *Brassica oleracea* identified eight genes with increased expression in elongating versus non-elongating hypocotyl flanks under both stimuli. All are members of auxin biosynthesis [GLYCOSIDE HYDROLASE 3.5 (GH3.5)], signaling (SAUR50), and response (EXPANSIN A1) (Esmon et al., 2006). In addition, all eight genes contain at least one consensus AUXIN RESPONSE FACTOR (ARF)-binding auxin response element and no auxin-induced expression was observed in an ARF7 mutant background.

Studies also show that gene regulations in response to gravitational or mechanical stimulations showed a great overlap. In whole *Arabidopsis* seedlings, 55 of the 141 identified genes, changed in abundance by gravitational stimulation, increased or decreased in transcript levels by mechanical stimulation (Moseyko et al., 2002). An even greater overlap between mechanical and gravitational stimulation was found in root apices, where 1730 genes were differentially regulated within 60 min of gravitropic or mechanical stimulation (Kimbrough et al., 2004). The alterations on the transcript level induced by both stimuli overlap by 96%. Many of the altered transcripts show increased or decreased levels in other abiotic and biotic stresses, too. They have functions as transcriptional regulators, in cell wall modification, as transporters, kinases, phosphatases, in hormone metabolism and in the cell cycle.

The first proteomic experiment to map changes in protein expression used 2D-GE and identified 16 alterations on the protein level in *A. thaliana* roots within 2 h of gravitational stimulation, some of them showing an altered abundance after 30 min (Kamada et al., 2005). Functional categories included Ca^2+^ signaling, cytoskeleton stability, energy production, TCA cycle and chaperone function. Furthermore, a shift in the apparent molecular weight of proteins due to gravitropic responses was observed that may be caused by an altered glycosylation pattern. One of those proteins with a change in molecular weight is the 20S PROTEASOME β-SUBUNIT E1. According to the authors, the chaperone HEAT SHOCK COGNATE 70-2 and the proteasomal subunit may regulate dynamic processes involved in the response to changes of the gravitational vector.

In order to identify proteins involved in early signaling events, e.g., the conversion from the biophysical signal of sedimentation of amyloplasts to a biochemical stimulus, a study on *Arabidopsis* shoots focused on the early perception and signaling events of plants subjected to 2 and 4 min of a deviation from the vertical. To identify less-/agravitropic signaling mutants, the authors performed plant reorientations at 4°C, an approach known as the gravity-persistent signal (GPS) approach (Wyatt et al., 2002). GPS blocks the asymmetric auxin transport resulting in a lack of gravitropic curvature. If transferred back to room temperature the plant regains a bending phenotype. Using GPS treatment, 82 alterations on the protein level after gravitational stimulation were identified (Schenck et al., 2013). Thirty-five percent of the differentially expressed proteins were predicted to localize to chloroplasts or plastids, consistent with the hypothesis that gravity-sensing is related to these organelles (Kiss et al., 1989).

Promising candidates that were identified as being an important part of the perception of the gravitational vector and subsequent signaling are HEAT SHOCK PROTEIN 81-1 and GLUTATHIONE S-TRANSFERASE PHI 9 (GSTF9) and GSTF20. HSP81-1 is involved in abiotic stress signaling, induced by Ca^2+^ signaling and may interact with J-domain containing proteins like ALTERED RESPONSE TO GRAVITY 1 and ARG1-LIKE 2 that are already known to show reduced gravitropism (Sedbrook et al., 1999; Guan et al., 2003). GSTF9 and GSTF20 may regulate the synthesis of plant hormones or subsequent signaling (Chen et al., 2007; Schenck et al., 2013).

The first transcriptomic and metabolomic approach was a combined treatment of gravi- and photostimulation (blue or red light) of *Arabidopsis* seedlings (Millar and Kiss, 2013). Despite the current methodical limitations in quantification of primary metabolites, the incorporation of metabolomic profiling into gravitational research was overdue, because this level is the last step prior to the physiological response. Gravity and light treatments led to shifts in amino acid pools (e.g., alanine, asparagine, glutamine, glycine, and isoleucine), decrease of sucrose and increase of hexoses, as well as decreased levels of secondary metabolites. Many of these altered primary metabolites are responsive to abiotic and biotic stresses, underlining the hypothesis of gravitropism and phototropism as exogenous stress stimuli of plants. As an example, changes in the pool of phenylalanine may lead to alterations in flavonoid biosynthesis. Those secondary metabolites are known to have an important function in the crosstalk between ethylene and auxin (Muday et al., 2012). On the transcript level, a 90° treatment for 24 h resulted in 339 alterations in gene abundance. Regulations on the transcript level could be correlated to changes on the metabolite level (Millar and Kiss, 2013). Increased levels of key enzymes of amino acid biosynthesis, THREONINE ALDOLASE 1 and GLUTAMINE-DEPENDENT ASPARAGINE SYNTHASE 1, explain the increase of corresponding metabolites. The altered abundance of carbohydrate metabolism enzymes explains a decrease in sucrose and increase in hexose sugars. Key enzymes of phenylpropanoid biosynthesis, e.g., CHALCONE SYNTHASE, are decreased in abundance.

Results of all studies indicate that changes of the influence of the gravitational vector lead to a general stress response in plants. Analyses on the proteomic and metabolomic level (Kamada et al., 2005; Millar and Kiss, 2013; Schenck et al., 2013) further support the hypothesis that an altered influence of the gravity vector is perceived as environmental stress (Millar and Kiss, 2013; Schenck et al., 2013), emphasizing the contribution of cytoskeleton, calcium signaling and chaperone function to plant’s gravitational response. In tissue specific transcriptomic studies, roots or opposing tissue flanks of the shoot, the importance of the phytohormone auxin is underlined. Already after 2 min alterations in auxin responsive genes were observed (Kimbrough et al., 2004). Furthermore, genes of auxin biosynthesis, signaling and response are differentially regulated (Esmon et al., 2006; Dong et al., 2013; Taniguchi et al., 2014). These findings support the widely accepted Cholodny/Went theory, suggesting auxin as a driving factor of tropic responses. A lack of an auxin response in whole seedlings may be caused by a higher dilution of auxin biosynthesis, signaling and response genes in whole seedling RNA, because the action of the phytohormone is restricted to some cell layers in specialized tissues.

## Simulated Microgravity and Spaceflight

Plants are potential components of future life support systems for manned space travels. For this, plant responses to the space environment, and in particular to microgravity, have to be studied (**Table 2**). Transcriptomic and proteomic studies have been performed toward this goal. In contrast to reorientation experiments, spaceflight and real microgravity experiments pose additional challenges. A suitable hardware providing optimal culture and illumination conditions has to be developed and further spaceflight-related effects on plants have to be taken into account such as increased levels of radiation, poor exchange of gases due to the lack of convection, vibrations, and accelerations depending on the transport systems and operations onboard (Porterfield et al., 1997).

**Table 2:** Studies that quantified differential expression of genes, proteins, and metabolites in response to simulated microgravity and spaceflight.

Plant age^1^	Tissue	Replicates	Method	Duration	DEG/DEP /DEM	Growth hardware	Treatment	Reference
**Transcriptomics**			
1 week	*A. thaliana* shoots	1	21K	5 days	SF/GC: 182	PGF, Petri dish, white light	STS93 (light)	Paul et al., 2005
Spores	*C. richardii* spores	4	custom array	1,8,20 h	SF/GC: 365	BRIC, Petri dish, white light	STS93 (light)	Salmi and Roux, 2008
6 days	*A. thaliana* seedlings	n.a.	44K	1 h, 6 days	1 h: 412 6 days: 502	Petri dish, white light	3D clinostat (light)	Soh et al., 2011
Seeds	A. *thaliana etiolated* seedlings and callus culture	6–7	ATH1	13 days	SF/GC: 300	BRIC-LED, Petri dish, dark	STS131 (dark)	Paul et al., 2012b
Seeds	*A. thaliana* leaves, roots, hypocotyls	3	ATH1	12 days	SF/GC: 480	ABRS, Petri dish, white light	ISS (light)	Paul et al., 2013
4 days	*A. thaliana* seedlings	2–3	ATH1	2 days	SF/GC: 230 SF/FC: 27	EMCS, TROPI-2, white light	ISS, (blue, red/blue light)	Correll et al., 2013
Seeds	*B. rapa* var. *nipposinica*	1	RNAseq	27 days	SF/GC: 22.428	LADA, white light	ISS (light)	Sugimoto et al., 2014
Seeds	*A. thaliana* seedlings	3	ATH1	14 days	SF/GC: 192	BRIC, Petri dish, dark	STS131	Kwon et al., 2015
11 days	*A. thaliana* callus cultures	2	ATH1	5 days	SF/GC: 9 SF/FC: 0	SIMBOX, 1.2a plates	Shenzou-8	Fengler et al., 2015
**Proteomics**			
1 week	*A. thaliana* callus culture	3	2D-GE	8 h	18	Petri dish with solid medium	2D clinostat	Wang et al., 2006
1 week	*A. thaliana* callus culture	3	2D-GE	2 h	Clinorotation: 19 RPM: 8	Tubes and Petri dish	2D clinostat, RPM	Barjaktarović et al., 2007
1 week	*A. thaliana* callus culture	3	2D-GE Pro-Q	30 min	24	Petri dish	RPM	Barjaktarović et al., 2009
4 days	*A. thaliana* root	8	2D-GE	12 h	23	Culture flask	2D clinostat (dark)	Tan et al., 2011
1 week	*A. thaliana* callus culture	3	2D-GE	200 min	Levitation: 60 RPM: 1	Petri dish	RPM (dark) Diamagnetic Levitation (dark)	Herranz et al., 2013b
Seeds	*A. thaliana* seedlings		Label free	12 days	SF/GC: 149	EMCS, GENARA A	ISS (light)	Mazars et al., 2014b
3 days	*A. thaliana* callus culture	2	iTRAQ	14 days	45	SIMBOX	SHENZOU-8	Zhang et al., 2015
Seeds	*A. thaliana* leaves and roots	1–3	iTRAQ	12 days	Leaf: 256 Root: 358	ABRS, solid media plates	ISS	Ferl et al., 2015
8 days	*A. thaliana* roots	3	2D-GE	30 min	μg: 6 90°: 1	Petri dish, white light	2D clinostat (dark)	Schüler et al., 2015
**Transcriptomics and Proteomics**			
1 week	*A. thaliana* callus culture	2–3	2D-GE Pro-Q ATH1	20 s	PF/FC: 12 DEP PF/FC: 881 DEG	Culture flask with solid medium	Parabolic flight	Hausmann et al., 2014

*^1^At start of treatment; DEG/DEP/DEM, differentially expressed genes, proteins, metabolites; n.a., information not available; Rep, number of biological replicates; ABRS, advanced biological research system; BRIC, biological research in canisters; BRIC-LED, biological research in a canister–light-emitting diode; EMCS, European Modular Cultivation System; FC, 1 g inflight control; GC, 1 g ground control; PF, parabolic flight; PRO-Q, Pro-Q diamond phosphoprotein-stain; RPM, random positioning machine; SF, spaceflight; STS, space transportation system.*

In order to avoid some of these additional environmental effects and allow a discrimination of pure microgravity-related effects, 1 g controls are essential. Under optimal conditions these are realized by the use of onboard 1 g reference centrifuges. It is, however, in most cases common to compare space flight samples to corresponding 1 g ground controls. Efforts have been made to replicate the growth conditions in space for the ground controls by using orbital environment simulators (OES) that replicate light, temperature and CO_2_ conditions as recorded in space. Studies utilizing both controls show that more alterations in gene expression are observed comparing plants from space flight (SF) to ground controls (GC) than to on board 1 g flight controls (FC) (Correll et al., 2013; Fengler et al., 2015), suggesting that factors independent of gravity and operational-induced side effects have a profound effect on plant growth and development.

Another consideration is the age of plants that are transported into orbit. Dried seeds do not correspond to environmental changes during the transport phase. Callus cultures and plants that are transported as seedlings or fully grown, however, experience transient changes in accelerations, 1 g on ground, hypergravity during flight phase and microgravity in orbit, and other changes in growth conditions that are not only a result of the space environment.

Different experimental conditions therefore have a great effect on the responses of plants to the space environment. In leaves of wheat, for example, no changes in gene expression were detectable between ground control and space-grown plants (Stutte et al., 2006). Growth of *Arabidopsis* seedlings during space flight though, led to the alteration of 480 genes compared to ground controls (Paul et al., 2013). The latter study was also the first one to study responses in different plant organs separately, indicating that different organs display unique patterns of gene expression in response to spaceflight. From a total of 480 genes that were differentially expressed in leaves, hypocotyls, or roots, only 26 genes were uniformly regulated in all three organs, many of those being involved in cell wall remodeling.

*Arabidopsis* plants grown in a spaceflight environment are usually smaller than ground controls, possess smaller roots and fewer lateral roots (Paul et al., 2012a). The cell wall of *Arabidopsis* and rice plants is reduced in response to the spaceflight environment (Hoson et al., 2002, 2003; Soga et al., 2002). Furthermore, cell wall extensibility of shoots is increased, while the cell wall flexibility of roots is decreased, as compared to ground controls (Hoson et al., 2003). Transcriptomic and proteomic approaches showed transcripts and proteins associated with cell wall remodeling, root hair generation and cell expansion to be highly altered during spaceflight (Paul et al., 2012b, 2013; Correll et al., 2013; Mazars et al., 2014b; Fengler et al., 2015; Ferl et al., 2015; Kwon et al., 2015; Zhang et al., 2015) or during clinorotation and random positioning (Wang et al., 2006; Barjaktarović et al., 2009). Alterations in gene expression that have a function in cell wall modification could be caused by spaceflight-induced changes in several hormone signaling pathways that are mediating growth and cell expansion. By using spaceflight 1 g controls and 1 g ground controls to elucidate the response to microgravity and exclude indirect effects by the spaceflight, 27 strictly graviresponsive transcripts were identified that were altered at least twofold in abundance, including genes of cell wall metabolism and actin cytoskeleton organization (Correll et al., 2013). By regulating the transport of cell wall components, the actin cytoskeleton is essential for a proper biosynthesis of the cell wall (Baluska et al., 2002). Also proteomic studies of *Arabidopsis* microsomes and callus cultures supported the involvement of cell wall modifications (Mazars et al., 2014b). A subsequent comparison of 1 g space control and 1 g ground controls of the same flight could furthermore show that cell wall modifying proteins are largely not altered on the protein level, suggesting that cell wall modifying enzymes are necessary for a response specifically to microgravity (Mazars et al., 2014a; Zhang et al., 2015). A comparison of different studies furthermore showed that regulation of protein activity occurs on multiple levels. REVERSIBLY GLYCOSYLATED POLYPEPTIDEs, involved in cell wall metabolism, did not only show a decreased abundance on the protein level (Wang et al., 2006; Schüler et al., 2015), but were also phosphorylated in response to clinorotation (Barjaktarović et al., 2009). Phosphorylation was suggested to affect the activity of the proteins and thereby cell wall metabolism.

In addition to changes in primary and lateral roots, *Arabidopsis* seedlings grown in space experience reduced root hair development (Kwon et al., 2015). This decrease may be due to reduced levels of peroxidases and cell wall modifying genes (Kwon et al., 2015). Out of 174 transcripts showing an altered abundance, 56 are enriched in root hairs and eight were shown to function in root hair development. Mutations in those peroxidases with a decreased abundance by spaceflight, led to a disruption of root hair formation in *Arabidopsis* (Kwon et al., 2015).

In other comparable spaceflight experiments, genes involved in production and response to ROS were altered (Paul et al., 2012b; Correll et al., 2013). A change in ROS levels is a secondary messenger of many abiotic and biotic stresses in plants (Apel and Hirt, 2004). ROS concentrations in the cell are kept in balance by an interplay of ROS production and scavenging via different enzymes and metabolites (Pitzschke and Hirt, 2006). Spaceflight/microgravity affects genes involved in ROS production and homeostasis by up- or downregulation. Increase in hydrogen peroxide levels and increased expression and phosphorylation of ROS scavengers, e.g., SUPEROXIDE DISMUTASE, CATALASE, GLUTATHIONE PEROXIDASE, THIOREDOXIN, and GLUTAREDOXIN, and marker genes were measured in short-term and long-term experiments (Hausmann et al., 2014; Sugimoto et al., 2014). Also in microgravity simulated by clinorotation, genes with antioxidant activity showed increased expression levels in response to short-term and long-term microgravity (Soh et al., 2011). In contrast, proteins of the response to oxidative stress were decreased in *Arabidopsis* calli and in *C. richardii* spores (Salmi and Roux, 2008; Zhang et al., 2015). This may indicate that different isoform of gene families are differentially regulated, as was directly observed in some studies (Barjaktarović et al., 2007; Fengler et al., 2015). ROS changes in plants might be a direct result of auxin signaling. In response to microgravity treatment of roots from wildtype and *pin2* mutant plants, a peroxidase was identified that showed altered levels in the wildtype, but not in the mutant (Tan et al., 2011).

In order to maintain ROS homeostasis for cell function and for signaling, proteins involved in this process are likely to be also posttranslationally regulated. Phosphoproteomic studies show a differential phosphorylation in response to 30 min of microgravity of proteins which are responsive to ROS (Barjaktarović et al., 2009). Taken together, these observations indicate that a complex regulation of antioxidant enzymes is necessary to maintain ROS homeostasis under microgravity, and that different levels of regulation are involved in this process.

Besides ROS, also transcripts involved in calcium signaling are altered under space conditions (Salmi and Roux, 2008; Soh et al., 2011; Paul et al., 2012b; Correll et al., 2013; Mazars et al., 2014b). Plants respond to a spaceflight environment with disruptions of calcium localization and signaling (Klymchuk et al., 2001; Nedukha et al., 2001; Salmi and Roux, 2008). Increase in calcium concentrations have been measured using genetically encoded reporters within 20 s of microgravity during parabolic flights (Hausmann et al., 2014). In addition, up to 25 calcium dependent genes were upregulated at the end of the 20 s microgravity phase. A proteomic approach focusing on microsomal membranes found further evidence for a link between calcium and auxin signaling in response to microgravity. PHOTOTROPIN 2 (PHOT2) is decreased in abundance after 12 days of space flight (Mazars et al., 2014b). PHOT2 is a blue light receptor and can trigger intracellular calcium increases. It was suggest that the calcium increase activates calcium sensors, such as TOUCH3 and PINOID BINDING PROTEIN 1 (PBP1) that interact with the AGC kinase PINOID (PID), a regulator of PAT (Mazars et al., 2014a). In the same experiment, TOUCH3 protein abundance was increased fourfold at the plasma membrane, indicating a calcium dependent regulation of PAT in response to microgravity. This hypothesis was further supported by decreased levels of CATION EXCHANGER 1, a protein of the vacuolar membrane that drives calcium influx from the cytosol to the vacuole, which leads to an increased cytosolic calcium concentration.

Taken together, these data point toward a similar response mechanism in microgravity as described for the reorientation of plants in the gravitational field. In microgravity, calcium is increased, thereby activating calcium binding proteins, leading to the activation of kinases, including CPK11 and PINOID. Calcium influx may directly trigger production of ROS, e.g., via the activation of calcium dependent protein kinases, or indirectly via changes in auxin transport within the plant.

Tissues involved in the perception of the gravitational stimulus, e.g., the root cap, express all proteins necessary for this signaling model. However, other cell types, e.g., undifferentiated cell cultures, are also able to detect a loss of the influence of gravity (microgravity) without specialized gravisensing tissue (Martzivanou et al., 2006). In these cells, altered ROS production in response to changes in the influence of the gravitational field might be a result of general, but tissue-specific stress responses (Wang et al., 2006; Barjaktarović et al., 2007, 2009, Salmi and Roux, 2008; Herranz et al., 2013b; Fengler et al., 2015). Also in whole seedlings subjected to simulated microgravity or spaceflight, transcripts of stress related genes are oftentimes altered (Kwon et al., 2015). Short-term microgravity environments, e.g., parabolic flights, thereby induce similar transcript changes, i.e., cell wall, heat shock, response to hormones, which are also observed after long term spaceflight experiments. Transcripts of functionally related genes were regulated in different tissues. Expression of individual isoforms, however, was specific to different plant organs (Paul et al., 2013). A comparison of *Arabidopsis* callus cultures and seedlings grown in the same hardware showed two independent responses to spaceflight without similarities in transcript alterations (Paul et al., 2012b). In seedlings as well as callus cultures a response to abiotic and biotic stress was clearly detectable, but more pronounced in cell cultures (Paul et al., 2012b). The response to heat shock is the most prominent Gene Ontology (GO) in cell cultures followed by a general stress response (Paul et al., 2012b; Zupanska et al., 2013). Also in *Arabidopsis* shoots changes in HSP transcripts were observed (Paul et al., 2005). When plant responses to changes of the orientation with respect to the gravitational vector or to changes of intensity of the gravitational field were analyzed in the same study, HSP70-3 was the only protein with altered levels under both conditions (Schüler et al., 2015). Taking into account that the spaceflight environment includes several abiotic stresses, e.g., radiation, microgravity, or vibrations, over-expression of heat shock proteins may contribute to generalized tolerance for multiple alterations in environment conditions and may help to maintain cytoskeletal architecture, cell shaping, and protein remodeling (Swindell et al., 2007; Zupanska et al., 2013).

The notion that microgravity may constitute an abiotic stress, led some authors to screen for genes involved in Simulated Microgravity Stress (SMS; Soh et al., 2011). SMS-genes including WRKY transcription factors and phytohormone induced signaling transcripts were identified. On this gene list, some transcripts are known to be responsive to other biotic/abiotic stresses, too. This is supported by a proteomic approach that identified 18 altered proteins in *Arabidopsis* cell culture after 8 h of 3D clinorotation (Wang et al., 2006). Seven alterations are involved in stress responses (e.g., ALDEHYDE DEHYDROGENASE 2, GST, and CHITINASE). Also in seedlings, proteins, with an altered abundance, and being involved in general stress responses, were identified (Mazars et al., 2014b).

In summary, experimental approaches to impose changes of the gravitational field vary significantly in experimental setup and plant material. However, some common conclusions can be drawn from these studies. The plant cell wall and actin cytoskeleton are major targets for modifications. Earlier studies clearly show a spaceflight-induced cell wall thinning (Hoson et al., 2002, 2003). Root hair growth is also highly reduced in space (Kwon et al., 2015). The authors suggest a role of ROS in the alteration in growth. Results from further studies suggest that cell wall modifications of plants subjected to spaceflight may be directed by changes to the actin cytoskeleton (Correll et al., 2013; Mazars et al., 2014b).

Another response to simulated microgravity and spaceflight seems to be a general response to abiotic and biotic stresses. Especially cell cultures show a heat shock response. Those chaperones may help to maintain cytoskeletal architecture and cell shaping in a spaceflight environment (Zupanska et al., 2013). Furthermore, the biosynthesis and response to phytohormones and calcium signaling is altered under simulated microgravity and spaceflight conditions supporting the hypothesis that a highly reduced gravity environment resembles an abiotic and/or biotic stress in plant tissues (Salmi and Roux, 2008; Soh et al., 2011; Correll et al., 2013; Mazars et al., 2014b). Another stress marker is the change in cellular ROS levels. In some of the reviewed publications an increase of ROS scavengers (Sugimoto et al., 2014), an elevated level in hydrogen peroxide (Hausmann et al., 2014) and an alteration of genes with antioxidant activity (Soh et al., 2011) was shown if plants were subjected to spaceflight or simulated microgravity. In addition, dependency of some responses on the PIN2 auxin transporter and changes in calcium concentrations point towards a signaling mechanism in microgravity, involving calcium, ROS and auxin, that is comparable to the response to a reorientation in the gravitational field.

## Conclusion/Future Perspectives

The reviewed studies identified molecular components of plant responses to changes in the gravitational field or vector. It was previously known that calcium and auxin play a role in these processes. Omics profiling strategies allowed the identification of underlying genes and proteins with altered abundance by these messengers or that affect the function of these messengers. Especially alterations in the concentration of auxin biosynthesis (GH3.5) and auxin responsive (AUX/IAA, SAUR) genes have been observed. A comparison of these studies furthermore indicates that different plant organs and callus cultures respond differently to changes in the influence of gravity. More cell type specific studies are necessary to identify how different cell types respond and how these cell types interact to form plant responses. Since experimental time in space is limited, experiments in ground-based facilities (GBFs) will be extensively needed to compensate for space experiments and to provide the necessary number of replicates for robust results.

Hardware-specific differences between individual studies as well as differential operations of GBFs contribute to difficulties in understanding plant responses to changes in the influence of the gravitational field. Standardization of growth and experimental hardware has to be pursued for ground-based and flight facilities (Schüler et al., 2015). Validated operational parameters of simulation approaches and 1 g flight control will have to be an essential component in all space flight experiments. The generation of more robust data would also benefit from scientific collaborations that perform the same experiments over multiple missions using standardized hardware. Sequential extraction protocols for RNA, proteins and metabolites would also reduce biological material and facilitate analyses across different levels of responses.

One of the main tasks for the future will be the integration of different datasets, covering various levels of cellular responses, ions, transcript changes, proteins, and hormones, into a common database to allow researchers to cross-analyse all results between different experimental conditions, tissues, and organisms. This will increase statistical power as compared to individual analyses with limited biological replicates. Larger datasets also allow for the development of mathematical models that are both descriptive and predictive and enable the generation of testable hypotheses. Complementary to broad omics techniques, reporter techniques with single cell resolution, e.g., genetically encoded hormone-, pH- and Ca^2+^-reporters, should be used to study signaling events and signal transmission, e.g., from the columella cells to the elongation zone. Suitable hardware to study single cell response to changes in the influence of the gravitational vector is available in the form of microscopes with a vertical sample stage. Microscopes for cell specific studies under microgravity conditions have also been made available.

Next logical steps after the development of testable mathematical models are physiological tests of the coding genes by experiments with mutant lines and overexpression lines. These are necessary to further corroborate their function in gravitational responses. Limitation in experiments with transgenic plants in space and ground-based facilities can be overcome with the application of chemically mutagenized lines or newer techniques, e.g., CRISPR/Cas.

## Conflict of Interest Statement

The authors declare that the research was conducted in the absence of any commercial or financial relationships that could be construed as a potential conflict of interest.

## References

[B1] Abas L. Benjamins R. Malenica N. Paciorek T. Wisniewska J. Moulinier-Anzola J. C. (2006). Intracellular trafficking and proteolysis of the *Arabidopsis* auxin-eﬄux facilitator PIN2 are involved in root gravitropism. *Nat. Cell Biol.* 8 249–256. 10.1038/ncb1369 16489343

[B2] Allen N. S. Chattaraj P. Collings D. Johannes E. (2003). Gravisensing: ionic responses, cytoskeleton and amyloplast behavior. *Adv. Space Res.* 32 1631–1637. 10.1016/s0273-1177(03)90404-90402 15015476

[B3] Apel K. Hirt H. (2004). Reactive oxygen species: metabolism, oxidative stress, and signal transduction. *Annu. Rev. Plant Biol.* 55 373–399. 10.1146/annurev.arplant.55.031903.141701 15377225

[B4] Baluska F. Hlavacka A. Samaj J. Palme K. Robinson D. G. Matoh T. (2002). F-actin-dependent endocytosis of cell wall pectins in meristematic root cells. Insights from brefeldin A-induced compartments. *Plant Physiol.* 130 422–431. 10.1104/pp.007526 12226521 PMC166574

[B5] Band L. R. Wells D. M. Larrieu A. Sun J. Middleton A. M. French A. P. (2012). Root gravitropism is regulated by a transient lateral auxin gradient controlled by a tipping-point mechanism. *Proc. Natl. Acad. Sci. U.S.A.* 109 4668–4673. 10.1073/pnas.1201498109 22393022 PMC3311388

[B6] Barbosa I. C. Zourelidou M. Willige B. C. Weller B. Schwechheimer C. (2014). D6 PROTEIN KINASE activates auxin transport-dependent growth and PIN-FORMED phosphorylation at the plasma membrane. *Dev. Cell* 29 674–685. 10.1016/j.devcel.2014.05.006 24930721

[B7] Barjaktarović Z. Nordheim A. Lamkemeyer T. Fladerer C. Madlung J. Hampp R. (2007). Time-course of changes in amounts of specific proteins upon exposure to hyper-g, 2-D clinorotation, and 3-D random positioning of *Arabidopsis* cell cultures. *J. Exp. Bot.* 58 4357–4363. 10.1093/jxb/erm302 18182437

[B8] Barjaktarović Ž. Schütz W. Madlung J. Fladerer C. Nordheim A. Hampp R. (2009). Changes in the effective gravitational field strength affect the state of phosphorylation of stress-related proteins in callus cultures of *Arabidopsis thaliana*. *J. Exp. Bot.* 60 779–789. 10.1093/jxb/ern324 19129159 PMC2652066

[B9] Barlow P. (1974). Regeneration of the cap of primary roots of *Zea mays*. *New Phytol.* 73 937–954. 10.1111/j.1469-8137.1974.tb01323.x.

[B10] Belyavskaya N. A. (1996). “Calcium and graviperception in plants: inhibitor analysis,” in *International Review of Cytology* ed. Kwang W. J. (Waltham, MA: Academic Press) 123–185.

[B11] Blakeslee J. J. Bandyopadhyay A. Lee O. R. Mravec J. Titapiwatanakun B. Sauer M. (2007). Interactions among PIN-FORMED and P-glycoprotein auxin transporters in *Arabidopsis*. *Plant Cell* 19 131–147. 10.1105/tpc.106.040782 17237354 PMC1820964

[B12] Blancaflor E. B. (2013). Regulation of plant gravity sensing and signaling by the actin cytoskeleton. *Am. J. Bot.* 100 143–152. 10.3732/ajb.1200283 23002165

[B13] Blancaflor E. B. Fasano J. M. Gilroy S. (1998). Mapping the functional roles of cap cells in the response of *arabidopsis* primary roots to gravity. *Plant Physiol.* 116 213–222. 10.1104/pp.116.1.213 9449842 PMC35160

[B14] Blancaflor E. B. Masson P. H. (2003). Plant gravitropism. Unraveling the ups and downs of a complex process. *Plant Physiol.* 133 1677–1690. 10.1104/pp.103.032169 14681531 PMC1540347

[B15] Braun M. Buchen B. Sievers A. (2002). Actomyosin-mediated statolith positioning in gravisensing plant cells studied in microgravity. *J. Plant Growth Regul.* 21 137–145. 10.1007/s003440010052 12016508

[B16] Braun M. Limbach C. (2006). Rhizoids and protonemata of characean algae: model cells for research on polarized growth and plant gravity sensing. *Protoplasma* 229 133–142. 10.1007/s00709-006-0208-209 17180494

[B17] Caspar T. Pickard B. G. (1989). Gravitropism in a starchless mutant of *Arabidopsis*: implications for the starch-statolith theory of gravity sensing. *Planta* 177 185–197. 10.1007/BF00392807 11539758

[B18] Chatterjee A. Porterfield D. M. Smith P. S. Roux S. J. (2000). Gravity-directed calcium current in germinating spores of *Ceratopteris richardii*. *Planta* 210 607–610. 10.1007/s004250050050 10787054

[B19] Chen I. C. Huang I. C. Liu M. J. Wang Z. G. Chung S. S. Hsieh H. L. (2007). Glutathione S-transferase interacting with far-red insensitive 219 is involved in phytochrome A-mediated signaling in *Arabidopsis*. *Plant Physiol.* 143 1189–1202. 10.1104/pp.106.094185 17220357 PMC1820923

[B20] Chen R. Hilson P. Sedbrook J. Rosen E. Caspar T. Masson P. H. (1998). The *Arabidopsis thaliana* AGRAVITROPIC 1 gene encodes a component of the polar-auxin-transport eﬄux carrier. *Proc. Natl. Acad. Sci. U.S.A.* 95 15112–15117. 10.1073/pnas.95.25.15112 9844024 PMC24584

[B21] Chen R. Rosen E. Masson P. H. (1999). Gravitropism in higher plants. *Plant Physiol.* 120 343–350. 10.1104/pp.120.2.343. 11541950 PMC1539215

[B22] Correll M. J. Pyle T. P. Millar K. D. Sun Y. Yao J. Edelmann R. E. (2013). Transcriptome analyses of *Arabidopsis thaliana* seedlings grown in space: implications for gravity-responsive genes. *Planta* 238 519–533. 10.1007/s00425-013-1909-x 23771594

[B23] Cowles J. R. Scheld H. W. Lemay R. Peterson C. (1984). Growth and lignification in seedlings exposed to eight days of microgravity. *Ann. Bot.* 54 33–48. 11539752 10.1093/oxfordjournals.aob.a086865

[B24] Dolan L. Janmaat K. Willemsen V. Linstead P. Poethig S. Roberts K. (1993). Cellular organisation of the *Arabidopsis thaliana* root. *Development* 119 71–84. 8275865 10.1242/dev.119.1.71

[B25] Dong Z. Jiang C. Chen X. Zhang T. Ding L. Song W. (2013). Maize LAZY1 mediates shoot gravitropism and inflorescence development through regulating auxin transport, auxin signaling, and light response. *Plant Physiol.* 163 1306–1322. 10.1104/pp.113.227314 24089437 PMC3813652

[B26] Esmon C. A. Tinsley A. G. Ljung K. Sandberg G. Hearne L. B. Liscum E. (2006). A gradient of auxin and auxin-dependent transcription precedes tropic growth responses. *Proc. Natl. Acad. Sci. U.S.A.* 103 236–241. 10.1073/pnas.0507127103 16371470 PMC1324985

[B27] Fasano J. M. Massa G. D. Gilroy S. (2002). Ionic signaling in plant responses to gravity and touch. *J. Plant Growth Regul.* 21 71–88. 10.1007/s003440010049 12016507

[B28] Fasano J. M. Swanson S. J. Blancaflor E. B. Dowd P. E. Kao T. H. Gilroy S. (2001). Changes in root cap pH are required for the gravity response of the *Arabidopsis* root. *Plant Cell* 13 907–921. 10.1105/tpc.13.4.907 11283344 PMC135544

[B29] Fengler S. Spirer I. Neef M. Ecke M. Nieselt K. Hampp R. (2015). A whole-genome microarray study of *Arabidopsis thaliana* semisolid callus cultures exposed to microgravity and nonmicrogravity related spaceflight conditions for 5 days on board of Shenzhou 8. *Biomed. Res. Int.* 2015:547495 10.1155/2015/547495 PMC4309294 25654111

[B30] Ferl R. J. Koh J. Denison F. Paul A.-L. (2015). Spaceflight induces specific alterations in the proteomes of *Arabidopsis*. *Astrobiology* 15 32–56. 10.1089/ast.2014.1210 25517942 PMC4290804

[B31] Frank A. B. (1868). “Über die durch die Schwerkraft verursachten Bewegungen von Pflanzentheilen,” in *Beiträge zur Pflanzenphysiologie* (Leipzig: Verlag von Wilhelm Engelmann) 1–99.

[B32] Friml J. (2003). Auxin transport - shaping the plant. *Curr. Opin. Plant Biol* 6 7–12. 10.1016/S1369526602000031 12495745

[B33] Friml J. Benkova E. Blilou I. Wisniewska J. Hamann T. Ljung K. (2002a). AtPIN4 mediates sink-driven auxin gradients and root patterning in *Arabidopsis*. *Cell* 108 661–673. 10.1016/S0092-8674(02)00656-6 11893337

[B34] Friml J. Wisniewska J. Benkova E. Mendgen K. Palme K. (2002b). Lateral relocation of auxin eﬄux regulator PIN3 mediates tropism in *Arabidopsis*. *Nature* 415 806–809. 10.1038/415806a 11845211

[B35] Fukaki H. Wysocka-Diller J. Kato T. Fujisawa H. Benfey P. N. Tasaka M. (1998). Genetic evidence that the endodermis is essential for shoot gravitropism in *Arabidopsis thaliana*. *Plant J.* 14 425–430. 10.1046/j.1365-313X.1998.00137.x 9670559

[B36] Ganguly A. Lee S. H. Cho H. T. (2012). Functional identification of the phosphorylation sites of *Arabidopsis* PIN-FORMED3 for its subcellular localization and biological role. *Plant J.* 71 810–823. 10.1111/j.1365-313X.2012.05030.x 22519832

[B37] Geldner N. Richter S. Vieten A. Marquardt S. Torres-Ruiz R. A. Mayer U. (2004). Partial loss-of-function alleles reveal a role for GNOM in auxin transport-related, post-embryonic development of *Arabidopsis*. *Development* 131 389–400. 10.1242/dev.00926 14681187

[B38] Grebe M. (2005). Growth by auxin: when a weed needs acid. *Science* 310 60–61. 10.1126/science.1119735 16210521

[B39] Guan C. Rosen E. S. Boonsirichai K. Poff K. L. Masson P. H. (2003). The ARG1-LIKE2 gene of *Arabidopsis* functions in a gravity signal transduction pathway that is genetically distinct from the PGM pathway. *Plant Physiol.* 133 100–112. 10.1104/pp.103.023358 12970478 PMC196584

[B40] Haberlandt G. (1900). Über die Perzeption des geotropischen Reizes. *Ber. Dtsch. Bot. Ges.* 18 261–272.

[B41] Häder D.-P. Richter P. Lebert M. (2006). Signal transduction in gravisensing of flagellates. *Signal Trans.* 6 422–431. 10.1002/sita.200600104

[B42] Hasenstein K. H. Evans M. L. (1988). Effects of cations on hormone transport in primary roots of *Zea mays*. *Plant Physiol.* 86 890–894. 10.1104/pp.86.3.890 11538240 PMC1054589

[B43] Hausmann N. Fengler S. Hennig A. Franz-Wachtel M. Hampp R. Neef M. (2014). Cytosolic calcium, hydrogen peroxide and related gene expression and protein modulation in *Arabidopsis thaliana* cell cultures respond immediately to altered gravitation: parabolic flight data. *Plant Biol.* 16 120–128. 10.1111/plb.12051 23870071

[B44] Heilmann I. Shin J. Huang J. Perera I. Y. Davies E. (2001). Transient dissociation of polyribosomes and concurrent recruitment of calreticulin and calmodulin transcripts in gravistimulated maize pulvini. *Plant Physiol.* 127 1193–1203. 10.1104/pp.010538 11706198 PMC129287

[B45] Herranz R. Anken R. Boonstra J. Braun M. Christianen P. C. De Geest M. (2013a). Ground-based facilities for simulation of microgravity: organism-specific recommendations for their use, and recommended terminology. *Astrobiology* 13 1–17. 10.1089/ast.2012.0876 23252378 PMC3549630

[B46] Herranz R. Manzano A. I. Van Loon J. J. Christianen P. C. Medina F. J. (2013b). Proteomic signature of *Arabidopsis* cell cultures exposed to magnetically induced hyper- and microgravity environments. *Astrobiology* 13 217–224. 10.1089/ast.2012.0883 23510084

[B47] Hoson T. Soga K. Mori R. Saiki M. Nakamura Y. Wakabayashi K. (2002). Stimulation of elongation growth and cell wall loosening in rice coleoptiles under microgravity conditions in space. *Plant Cell Physiol.* 43 1067–1071. 10.1093/pcp/pcf126 12354926

[B48] Hoson T. Soga K. Wakabayashi K. Kamisaka S. Tanimoto E. (2003). Growth and cell wall changes in rice roots during spaceflight. *Plant Soil* 255 19–26. 10.1023/A:1026105431505 14631940

[B49] Hou G. Kramer V. L. Wang Y. S. Chen R. Perbal G. Gilroy S. (2004). The promotion of gravitropism in *Arabidopsis* roots upon actin disruption is coupled with the extended alkalinization of the columella cytoplasm and a persistent lateral auxin gradient. *Plant J.* 39 113–125. 10.1111/j.1365-313X.2004.02114.x 15200646

[B50] Hou G. Mohamalawari D. R. Blancaflor E. B. (2003). Enhanced gravitropism of roots with a disrupted cap actin cytoskeleton. *Plant Physiol.* 131 1360–1373. 10.1104/pp.014423 12644685 PMC166895

[B51] Hu L. Mei Z. Zang A. Chen H. Dou X. Jin J. (2013). Microarray analyses and comparisons of upper or lower flanks of rice shoot base preceding gravitropic bending. *PLoS ONE* 8:e74646 10.1371/journal.pone.0074646 PMC3764065 24040303

[B52] Huang F. Zago M. K. Abas L. Van Marion A. Galvan-Ampudia C. S. Offringa R. (2010). Phosphorylation of conserved PIN motifs directs *Arabidopsis* PIN1 polarity and auxin transport. *Plant Cell* 22 1129–1142. 10.1105/tpc.109.072678 20407025 PMC2879764

[B53] Ingber D. E. (1997). Tensegrity: the architectural basis of cellular mechanotransduction. *Annu. Rev. Physiol.* 59 575–599. 10.1146/annurev.physiol.59.1.575 9074778

[B54] Joo J. H. Bae Y. S. Lee J. S. (2001). Role of auxin-induced reactive oxygen species in root gravitropism. *Plant Physiol.* 126 1055–1060. 10.1104/pp.126.3.1055 11457956 PMC116462

[B55] Kamada M. Higashitani A. Ishioka N. (2005). Proteomic analysis of *Arabidopsis* root gravitropism. *Biol. Sci. Space* 19 148–154. 10.2187/bss.19.148

[B56] Kimbrough J. M. Salinas-Mondragon R. Boss W. F. Brown C. S. Sederoff H. W. (2004). The fast and transient transcriptional network of gravity and mechanical stimulation in the *Arabidopsis* root apex. *Plant Physiol.* 136 2790–2805. 10.1104/pp.104.044594 15347791 PMC523342

[B57] Kiss J. Z. (2000). Mechanisms of the early phases of plant gravitropism. *CRC Crit. Rev. Plant Sci.* 19 551–573. 10.1080/07352680091139295 11806421

[B58] Kiss J. Z. Guisinger M. M. Miller A. J. Stackhouse K. S. (1997). Reduced gravitropism in hypocotyls of starch-deficient mutants of *Arabidopsis*. *Plant Cell Physiol.* 38 518–525. 10.1093/oxfordjournals.pcp.a029199 9210329

[B59] Kiss J. Z. Hertel R. Sack F. D. (1989). Amyloplasts are necessary for full gravitropic sensitivity in roots of *Arabidopsis thaliana*. *Planta* 177 198–206. 10.1007/BF00392808 11539759

[B60] Kiss J. Z. Sack F. D. (1989). Reduced gravitropic sensitivity in roots of a starch-deficient mutant of *Nicotiana sylvestris*. *Planta* 180 123–130. 10.1007/BF02411418 11540920

[B61] Kleine-Vehn J. Ding Z. Jones A. R. Tasaka M. Morita M. T. Friml J. (2010). Gravity-induced PIN transcytosis for polarization of auxin fluxes in gravity-sensing root cells. *Proc. Natl. Acad. Sci. U.S.A.* 51 22344–22349. 10.1073/pnas.1013145107 21135243 PMC3009804

[B62] Kleine-Vehn J. Friml J. (2008). Polar targeting and endocytic recycling in auxin-dependent plant development. *Annu. Rev. Cell Dev. Biol.* 24 447–473. 10.1146/annurev.cellbio.24.110707.175254 18837671

[B63] Klymchuk D. O. Brown C. S. Chapman D. K. Vorobyova T. V. Martyn G. M. (2001). Cytochemical localization of calcium in soybean root cap cells in microgravity. *Adv. Space Res.* 27 967–972. 10.1016/S0273-1177(01)00160-0 11596641

[B64] Kuznetsov O. A. Hasenstein K. H. (1996). Intracellular magnetophoresis of amyloplasts and induction of root curvature. *Planta* 198 87–94. 10.1007/BF00197590 8580774

[B65] Kwon T. Sparks J. A. Nakashima J. Allen S. N. Tang Y. Blancaflor E. B. (2015). Transcriptional response of *Arabidopsis* seedlings during spaceflight reveals peroxidase and cell wall remodeling genes associated with root hair development. *Am. J. Bot.* 102 21–35. 10.3732/ajb.1400458 25587145

[B66] Leitz G. Kang B. H. Schoenwaelder M. E. Staehelin L. A. (2009). Statolith sedimentation kinetics and force transduction to the cortical endoplasmic reticulum in gravity-sensing *Arabidopsis columella* cells. *Plant Cell* 21 843–860. 10.1105/tpc.108.065052 19276442 PMC2671718

[B67] Limbach C. Hauslage J. Schafer C. Braun M. (2005). How to activate a plant gravireceptor. Early mechanisms of gravity sensing studied in characean rhizoids during parabolic flights. *Plant Physiol.* 139 1030–1040. 10.1104/pp.105.068106 16183834 PMC1256015

[B68] Löfke C. Zwiewka M. Heilmann I. Van Montagu M. C. Teichmann T. Friml J. (2013). Asymmetric gibberellin signaling regulates vacuolar trafficking of PIN auxin transporters during root gravitropism. *Proc. Natl. Acad. Sci. U.S.A.* 110 3627–3632. 10.1073/pnas.1300107110 23391733 PMC3587205

[B69] Lopez D. Tocquard K. Venisse J. S. Legue V. Roeckel-Drevet P. (2014). Gravity sensing, a largely misunderstood trigger of plant orientated growth. *Front. Plant Sci.* 5:610 10.3389/fpls.2014.00610 PMC4220637 25414717

[B70] Luschnig C. Gaxiola R. A. Grisafi P. Fink G. R. (1998). EIR1, a root-specific protein involved in auxin transport, is required for gravitropism in *Arabidopsis thaliana*. *Genes Dev.* 12 2175–2187. 10.1101/gad.12.14.2175 9679062 PMC317016

[B71] MacCleery S. A. Kiss J. Z. (1999). Plastid sedimentation kinetics in roots of wild-type and starch-deficient mutants of *Arabidopsis*. *Plant Physiol.* 120 183–192. 10.1104/pp.120.1.183 10318696 PMC59250

[B72] Mahonen A. P. Ten Tusscher K. Siligato R. Smetana O. Diaz-Trivino S. Salojarvi J. (2014). PLETHORA gradient formation mechanism separates auxin responses. *Nature* 515 125—129. 10.1038/nature13663 PMC4326657 25156253

[B73] Martzivanou M. Babbick M. Cogoli-Greuter M. Hampp R. (2006). Microgravity-related changes in gene expression after short-term exposure of *Arabidopsis thaliana* cell cultures. *Protoplasma* 229 155–162. 10.1007/s00709-006-0203-201 17180497

[B74] Mazars C. Briere C. Grat S. Pichereaux C. Rossignol M. Pereda-Loth V. (2014a). Microsome-associated proteome modifications of *Arabidopsis* seedlings grown on board the International Space Station reveal the possible effect on plants of space stresses other than microgravity. *Plant Signal. Behav.* 9:e29637 10.4161/psb.29637 PMC4205128 25763699

[B75] Mazars C. Brière C. Grat S. Pichereaux C. Rossignol M. Pereda-Loth V. (2014b). Microgravity induces changes in microsome-associated proteins of arabidopsis seedlings grown on board the international space station. *PLoS ONE* 9:e91814 10.1371/journal.pone.0091814 PMC3950288 24618597

[B76] McClure B. A. Guilfoyle T. (1989). Rapid redistribution of auxin-regulated RNAs during gravitropism. *Science* 243 91–93. 10.1126/science.11540631 11540631

[B77] Medina F. J. Herranz R. (2010). Microgravity environment uncouples cell growth and cell proliferation in root meristematic cells: the mediator role of auxin. *Plant Signal. Behav.* 5 176–179. 10.4161/psb.5.2.10966 20173415 PMC2884128

[B78] Millar K. D. Kiss J. Z. (2013). Analyses of tropistic responses using metabolomics. *Am. J. Bot.* 100 79–90. 10.3732/ajb.1200316 23196394

[B79] Morita M. T. (2010). Directional gravity sensing in gravitropism. *Annu. Rev. Plant Biol.* 61 705–720. 10.1146/annurev.arplant.043008.092042 19152486

[B80] Moseyko N. Zhu T. Chang H. S. Wang X. Feldman L. J. (2002). Transcription profiling of the early gravitropic response in *Arabidopsis* using high-density oligonucleotide probe microarrays. *Plant Physiol.* 130 720–728. 10.1104/pp.009688 12376639 PMC166601

[B81] Muday G. K. Rahman A. Binder B. M. (2012). Auxin and ethylene: collaborators or competitors? *Trends Plant Sci* 17 181–195. 10.1016/j.tplants.2012.02.001 22406007

[B82] Nedukha O. M. Kordyum E. L. Brown C. Chapman D. (2001). The interaction of microgravity and ethylene on the ultrastructure cell and Ca^2+^ localization in soybean hook hypocotyl. *J. Gravit. Physiol.* 8 P49–P50. 12638620

[B83] Ottenschlager I. Wolff P. Wolverton C. Bhalerao R. P. Sandberg G. Ishikawa H. (2003). Gravity-regulated differential auxin transport from columella to lateral root cap cells. *Proc. Natl. Acad. Sci. U.S.A.* 100 2987–2991. 10.1073/pnas.0437936100 12594336 PMC151453

[B84] Paciorek T. Zazimalova E. Ruthardt N. Petrasek J. Stierhof Y. D. Kleine-Vehn J. (2005). Auxin inhibits endocytosis and promotes its own eﬄux from cells. *Nature* 435 1251–1256. 10.1038/nature03633 15988527

[B85] Paudyal R. Jamaluddin A. Warren J. P. Doyle S. M. Robert S. Warriner S. L. (2014). Trafficking modulator TENin1 inhibits endocytosis, causes endomembrane protein accumulation at the pre-vacuolar compartment and impairs gravitropic response in *Arabidopsis thaliana*. *Biochem. J.* 460 177–185. 10.1042/bj20131136 24654932 PMC4100570

[B86] Paul A. L. Amalfitano C. E. Ferl R. J. (2012a). Plant growth strategies are remodeled by spaceflight. *BMC Plant Biol.* 12:232 10.1186/1471-2229-12-232 PMC3556330 23217113

[B87] Paul A. L. Zupanska A. K. Ostrow D. T. Zhang Y. Sun Y. Li J. L. (2012b). Spaceflight transcriptomes: unique responses to a novel environment. *Astrobiology* 12 40–56. 10.1089/ast.2011.0696 22221117 PMC3264962

[B88] Paul A.-L. Popp M. P. Gurley W. B. Guy C. Norwood K. L. Ferl R. J. (2005). *Arabidopsis* gene expression patterns are altered during spaceflight. *Adv. Space Res.* 36 1175–1181. 10.1016/j.asr.2005.03.066

[B89] Paul A.-L. Zupanska A. Schultz E. Ferl R. (2013). Organ-specific remodeling of the *Arabidopsis* transcriptome in response to spaceflight. *BMC Plant Biol.* 13:112 10.1186/1471-2229-13-112 PMC3750915 23919896

[B90] Perbal G. (1999). Gravisensing in roots. *Adv. Space Res.* 24 723–729. 10.1016/S0273-1177(99)00405-6 11542615

[B91] Perbal G. Driss-Ecole D. (2003). Mechanotransduction in gravisensing cells. *Trends Plant Sci.* 8 498–504. 10.1016/j.tplants.2003.09.005 14557047

[B92] Perera I. K. Heilmann I. Chang S. C. Boss W. F. Kaufman P. B. (2001a). A role for inositol 1,4,5-trisphosphate in gravitropic signaling and the retention of cold-perceived gravistimulation of oat shoot pulvini. *Plant Physiol.* 125 1499–1507. 10.1104/pp.125.3.1499 11244128 PMC65627

[B93] Perera I. Y. Heilmann I. Chang S. C. Boss W. F. Kaufman P. B. (2001b). A role for inositol 1,4,5-trisphosphate in gravitropic signaling and the retention of cold-perceived gravistimulation of oat shoot pulvini. *Plant Physiol.* 125 1499–1507. 10.1104/pp.125.3.1499 11244128 PMC65627

[B94] Perera I. Y. Heilmann I. Boss W. F. (1999). Transient and sustained increases in inositol 1,4,5-trisphosphate precede the differential growth response in gravistimulated maize pulvini. *Proc. Natl. Acad. Sci. U.S.A.* 96 5838–5843. 10.1073/pnas.96.10.5838 10318971 PMC21947

[B95] Pitzschke A. Hirt H. (2006). Mitogen-Activated protein kinases and reactive oxygen species signaling in plants. *Plant Physiol.* 141 351–356. 10.1104/pp.106.079160 16760487 PMC1475449

[B96] Plieth C. Trewavas A. J. (2002). Reorientation of seedlings in the earth’s gravitational field induces cytosolic calcium transients. *Plant Physiol.* 129 786–796. 10.1104/pp.011007 12068119 PMC161701

[B97] Porterfield D. M. (2002). The biophysical limitations in physiological transport and exchange in plants grown in microgravity. *J Plant Growth Regul.* 21 177–190. 10.1007/s003440010054 12024222

[B98] Porterfield D. M. Matthews S. W. Daugherty C. J. Musgrave M. E. (1997). Spaceflight exposure effects on transcription, activity, and localization of alcohol dehydrogenase in the roots of *Arabidopsis thaliana*. *Plant Physiol.* 113 685–693. 10.1104/pp.113.3.685 9085569 PMC158186

[B99] Sack F. D. (1991). Plant gravity sensing. *Int. Rev. Cytol.* 127 193–252. 10.1016/S0074-7696(08)60695-6 11536485

[B100] Sack F. D. (1997). Plastids and gravitropic sensing. *Planta* 203 S63–S68. 10.1007/Pl00008116 11540330

[B101] Salmi M. Ul Haque A. Bushart T. Stout S. Roux S. Porterfield D. M. (2011). Changes in gravity rapidly alter the magnitude and direction of a cellular calcium current. *Planta* 233 911–920. 10.1007/s00425-010-1343-1342 21234599

[B102] Salmi M. L. Roux S. J. (2008). Gene expression changes induced by space flight in single-cells of the fern *Ceratopteris richardii*. *Planta* 229 151–159. 10.1007/s00425-008-0817-y 18807069

[B103] Sato E. M. Hijazi H. Bennett M. J. Vissenberg K. Swarup R. (2015). New insights into root gravitropic signalling. *J. Exp. Bot.* 66 2155–2165. 10.1093/jxb/eru515 25547917 PMC4986716

[B104] Schenck C. A. Nadella V. Clay S. L. Lindner J. Abrams Z. Wyatt S. E. (2013). A proteomics approach identifies novel proteins involved in gravitropic signal transduction. *Am. J. Bot.* 100 194–202. 10.3732/ajb.1200339 23281391

[B105] Schüler O. Krause L. Görög M. Hauslage J. Kesseler L. Böhmer M. (2015). ARADISH - development of a standardized plant growth chamber for experiments in gravitational biology using ground based facilities. *Microgr. Sci. Technol.* 1–9. 10.1007/s12217-015-9454-9459.

[B106] Sedbrook J. C. Chen R. J. Masson P. H. (1999). ARG1 (Altered Response to Gravity) encodes a DnaJ-like protein that potentially interacts with the cytoskeleton. *Proc. Natl. Acad. Sci. U.S.A.* 96 1140–1145. 10.1073/pnas.96.3.1140 9927707 PMC15364

[B107] Sievers A. Buchen B. Volkmann D. Hejnowicz Z. (1991). “Role of the cytoskeleton in gravity perception,” in *The Cytoskeletal Basis of Plant Growth and Form* ed. Lloyd C. W. (London: Academic Press) 169–182.

[B108] Sinclair W. Trewavas A. J. (1997). Calcium in gravitropism. A re-examination. *Planta* 203 S85–S90. 10.1007/PL00008120 11540333

[B109] Soga K. Wakabayashi K. Kamisaka S. Hoson T. (2002). Stimulation of elongation growth and xyloglucan breakdown in *Arabidopsis* hypocotyls under microgravity conditions in space. *Planta* 215 1040–1046. 10.1007/s00425-002-0838-x 12355165

[B110] Soga K. Wakabayashi K. Kamisaka S. Hoson T. (2003). Growth restoration in azuki bean and maize seedlings by removal of hypergravity stimuli. *Adv. Space Res.* 31 2269–2274. 10.1016/S0273-1177(03)00254-0 14686442

[B111] Soh H. Auh C. Soh W.-Y. Han K. Kim D. Lee S. (2011). Gene expression changes in *Arabidopsis* seedlings during short- to long-term exposure to 3-D clinorotation. *Planta* 234 255–270. 10.1007/s00425-011-1395-y 21416242

[B112] Stanga J. P. Boonsirichai K. Sedbrook J. C. Otegui M. S. Masson P. H. (2009). A role for the TOC complex in *Arabidopsis* root gravitropism. *Plant Physiol.* 149 1896–1905. 10.1104/pp.109.135301 19211693 PMC2663755

[B113] Staves M. P. (1997). Cytoplasmic streaming and gravity sensing in Chara internodal cells. *Planta* 203 S79–S84. 10.1007/pl00008119 11540332

[B114] Staves M. P. Wayne R. Leopold A. C. (1997). The effect of the external medium on the gravitropic curvature of rice (*Oryza sativa*, Poaceae) roots. *Am. J. Bot.* 84 1522–1529. 10.2307/2446613 11541059

[B115] Stinemetz C. L. Kuzmanoff K. M. Evans M. L. Jarrett H. W. (1987). Correlation between calmodulin activity and gravitropic sensitivity in primary roots of maize. *Plant Physiol.* 84 1337–1342. 10.1104/pp.84.4.1337 11539677 PMC1056775

[B116] Strohm A. K. Barrett-Wilt G. A. Masson P. H. (2014). A functional TOC complex contributes to gravity signal transduction in *Arabidopsis*. *Front. Plant Sci.* 5:148 10.3389/fpls.2014.00148 PMC4001062 24795735

[B117] Stutte G. Monje O. Hatfield R. Paul A. Ferl R. Simone C. (2006). Microgravity effects on leaf morphology, cell structure, carbon metabolism and mRNA expression of dwarf wheat. *Planta* 224 1038–1049. 10.1007/s00425-006-0290-4 16708225

[B118] Sugimoto M. Oono Y. Gusev O. Matsumoto T. Yazawa T. Levinskikh M. A. (2014). Genome-wide expression analysis of reactive oxygen species gene network in Mizuna plants grown in long-term spaceflight. *BMC Plant Biol* 14:4 10.1186/1471-2229-14-14 PMC3927260 24393219

[B119] Sukumar P. Edwards K. S. Rahman A. Delong A. Muday G. K. (2009). PINOID kinase regulates root gravitropism through modulation of PIN2-dependent basipetal auxin transport in *Arabidopsis*. *Plant Physiol.* 150 722–735. 10.1104/pp.108.131607 19363095 PMC2689958

[B120] Swarup R. Friml J. Marchant A. Ljung K. Sandberg G. Palme K. (2001). Localization of the auxin permease AUX1 suggests two functionally distinct hormone transport pathways operate in the *Arabidopsis* root apex. *Genes Dev.* 15 2648–2653. 10.1101/gad.210501 11641271 PMC312818

[B121] Swindell W. R. Huebner M. Weber A. P. (2007). Transcriptional profiling of *Arabidopsis* heat shock proteins and transcription factors reveals extensive overlap between heat and non-heat stress response pathways. *BMC Genomics* 8:125 10.1186/1471-2164-8-125 PMC1887538 17519032

[B122] Tan C. Wang H. Zhang Y. Qi B. Xu G. Zheng H. (2011). A proteomic approach to analyzing responses of *Arabidopsis thaliana* root cells to different gravitational conditions using an agravitropic mutant, pin2 and its wild type. *Proteome Sci.* 9 72 10.1186/1477-5956-9-72 PMC3228730 22085406

[B123] Taniguchi M. Nakamura M. Tasaka M. Morita M. T. (2014). Identification of gravitropic response indicator genes in *Arabidopsis* inflorescence stems. *Plant Signal. Behav.* 9:e29570 10.4161/psb.29570 PMC4203507 25763694

[B124] Toyota M. Furuichi T. Tatsumi H. Sokabe M. (2008). *Cytoplasmic calcium* increases in response to changes in the gravity vector in hypocotyls and petioles of *Arabidopsis* seedlings. *Plant Physiol.* 146 505–514. 10.1104/pp.107.106450 18055589 PMC2245848

[B125] Valster A. H. Blancaflor E. B. (2008). “Mechanisms of gravity perception in higher plants,” in *Plant Tropisms* (Hoboken, MA: Blackwell Publishing Ltd) 3–19.

[B126] Volkmann D. Baluska F. (1999). Actin cytoskeleton in plants: from transport networks to signaling networks. *Microsc. Res. Technol.* 47 135–154. 10.1002/sic1097-0029 10523792

[B127] Wang H. Zheng H. Q. Sha W. Zeng R. Xia Q. C. (2006). A proteomic approach to analysing responses of *Arabidopsis thaliana* callus cells to clinostat rotation. *J. Exp. Bot.* 57 827–835. 10.1093/Jxb/Erj066 16449375

[B128] Wayne R. Staves M. P. (1996). A down to earth model of gravisensing or Newton’s Law of Gravitation from the apple’s perspective. *Physiol. Plant.* 98 917–921. 10.1111/j.1399-3054.1996.tb06703.x 11539338

[B129] Wayne R. Staves M. P. Leopold A. C. (1990). Gravity-dependent polarity of cytoplasmic streaming in Nitellopsis. *Protoplasma* 155 43–57. 10.1007/BF01322614 11540068

[B130] Went F. Thimann K. (1937). *Phytohormones* (New York, NY: Macmillan).

[B131] Whitford R. Fernandez A. Tejos R. Perez A. C. Kleine-Vehn J. Vanneste S. (2012). GOLVEN secretory peptides regulate auxin carrier turnover during plant gravitropic responses. *Dev. Cell* 22 678–685. 10.1016/j.devcel.2012.02.002 22421050

[B132] Wyatt S. E. Rashotte A. M. Shipp M. J. Robertson D. Muday G. K. (2002). Mutations in the Gravity persistence signal loci in *arabidopsis* disrupt the perception and/or signal transduction of gravitropic stimuli. *Plant Physiol.* 130 1426–1435. 10.1104/pp.102.010579 12428007 PMC166661

[B133] Yoder T. L. Zheng H. Q. Todd P. Staehelin L. A. (2001). Amyloplast sedimentation dynamics in maize columella cells support a new model for the gravity-sensing apparatus of roots. *Plant Physiol.* 125 1045–1060. 10.1104/pp.125.2.1045 11161060 PMC64904

[B134] Zadnikova P. Smet D. Zhu Q. Van Der Straeten D. Benkova E. (2015). Strategies of seedlings to overcome their sessile nature: auxin in mobility control. *Front. Plant Sci.* 6:218 10.3389/fpls.2015.00218 PMC4396199 25926839

[B135] Zhang Y. Wang L. Xie J. Zheng H. (2015). Differential protein expression profiling of *Arabidopsis thaliana* callus under microgravity on board the Chinese SZ-8 spacecraft. *Planta* 241 475–488. 10.1007/s00425-014-2196-x 25374148

[B136] Zheng H. Q. Staehelin L. A. (2001). Nodal endoplasmic reticulum, a specialized form of endoplasmic reticulum found in gravity-sensing root tip columella cells. *Plant Physiol.* 125 252–265. 10.1104/pp.125.1.252 11154334 PMC61007

[B137] Zupanska A. Denison F. Ferl R. Paul A.-L. (2013). Spaceflight engages heat shock protein and other molecular chaperone genes in tissue culture cells of *Arabidopsis thaliana*. *Am. J. Bot.* 100 235–248. 10.3732/ajb.1200343 23258370

